# Supplementary material for: Splice-Junction-Based Mapping of Alternative Isoforms in the Human Proteome
Source: Cell Rep. Author manuscript; Available in PMC 2020 Jan 15. (PMC6961840; doi:10.1016/j.celrep.2019.11.026)
Supplement: 2 [file NIHMS1546469-supplement-2.zip › DF1/PXD010154/Heart/Heart_42_MYCBP2_SLLVPVDNIFNASQGVR.pdf]

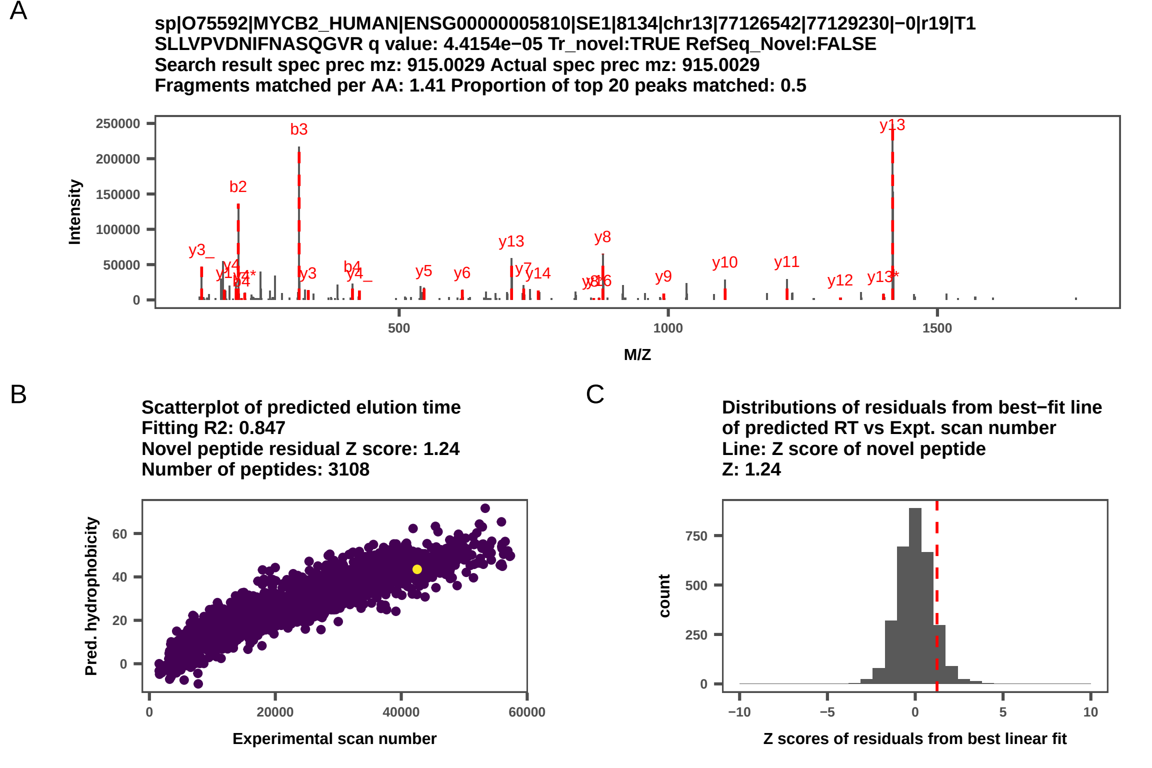
A
sp|O75592|MYCB2_HUMAN|ENSG00000005810|SE1|8134|chr13|77126542|77129230|−0|r19|T1
SLLVPVDNIFNASQGVR q value: 4.4154e−05 Tr_novel:TRUE RefSeq_Novel:FALSE
Search result spec prec mz: 915.0029 Actual spec prec mz: 915.0029
Fragments matched per AA: 1.41 Proportion of top 20 peaks matched: 0.5
Intensity
250000
200000
150000
100000
50000
0
b2
b3
y3_
y3
b4
y4_
y5
y6
y13
y7
y14
y8
y9
y10
y11
y12
y13
y13*
500
1000
1500
M/Z
B
Scatterplot of predicted elution time
Fitting R2: 0.847
Novel peptide residual Z score: 1.24
Number of peptides: 3108
Pred. hydrophobicity
60
40
20
0
0
20000
40000
60000
Experimental scan number
C
Distributions of residuals from best−fit line
of predicted RT vs Expt. scan number
Line: Z score of novel peptide
Z: 1.24
count
750
500
250
0
−10
−5
0
5
10
Z scores of residuals from best linear fit
